# A Decision-Tree Approach to Assist in Forecasting the Outcomes of the Neonatal Brain Injury

**DOI:** 10.3390/ijerph18094807

**Published:** 2021-04-30

**Authors:** Bogdan Mihai Neamțu, Gabriela Visa, Ionela Maniu, Maria Livia Ognean, Rubén Pérez-Elvira, Andrei Dragomir, Maria Agudo, Ciprian Radu Șofariu, Mihaela Gheonea, Antoniu Pitic, Remus Brad, Claudiu Matei, Minodora Teodoru, Ciprian Băcilă

**Affiliations:** 1 Clinical Department, Faculty of Medicine, Lucian Blaga University Sibiu, 550169 Sibiu, Romania; bogdan.neamtu@ulbsibiu.ro (B.M.N.); liviasibiu@gmail.com (M.L.O.); minodora.teodoru@ulbsibiu.ro (M.T.); 2 Department of Computer Science and Electrical Engineering, Faculty of Engineering, Lucian Blaga University Sibiu, 550025 Sibiu, Romania; antoniu.pitic@ulbsibiu.ro (A.P.); remus.brad@ulbsibiu.ro (R.B.); 3 Research and Telemedicine Center in Pediatric Neurology, Pediatric Clinical Hospital Sibiu, 550166 Sibiu, Romania; gabiap@yahoo.com (G.V.); ionela.maniu@ulbsibiu.ro (I.M.); andrei.drag@gmail.com (A.D.); ciprianradusofariu@gmail.com (C.R.S.); 4 Department of Mathematics and Informatics, Faculty of Sciences, Lucian Blaga University Sibiu, 550012 Sibiu, Romania; 5 Neonatology Department, Sibiu Clinical and Emergency County Hospital, Lucian Blaga University Sibiu, 550245 Sibiu, Romania; 6 Neuropsychophysiology Lab., NEPSA Rehabilitación Neurológica, 37003 Salamanca, Spain; mjagudojuan@gmail.com; 7 Biological and Health Psychology Department, Universidad Autónoma de Madrid, 280048 Madrid, Spain; 8 The N.1 Institute for Health, National University of Singapore, 28, Medical Dr. #05-COR, Singapore 117456, Singapore; 9 Neonatology Department, Craiova Clinical and Emergency County Hospital, University of Medicine and Pharmacy of Craiova, 200349 Craiova, Romania; drmgheonea@yahoo.com; 10 Dental and Nursing Medical Department, Faculty of Medicine, Lucian Blaga University Sibiu, 550169 Sibiu, Romania; claumatei@yahoo.com (C.M.); bacila_c@yahoo.com (C.B.); 11 Dr. Gheorghe Preda Psychiatric Hospital, 550082 Sibiu, Romania

**Keywords:** neonatal brain injury, risk factors, abnormal outcomes, seizures, neurodevelopment, decision-tree algorithms

## Abstract

Neonatal brain injury or neonatal encephalopathy (NE) is a significant morbidity and mortality factor in preterm and full-term newborns. NE has an incidence in the range of 2.5 to 3.5 per 1000 live births carrying a considerable burden for neurological outcomes such as epilepsy, cerebral palsy, cognitive impairments, and hydrocephaly. Many scoring systems based on different risk factor combinations in regression models have been proposed to predict abnormal outcomes. Birthweight, gestational age, Apgar scores, pH, ultrasound and MRI biomarkers, seizures onset, EEG pattern, and seizure duration were the most referred predictors in the literature. Our study proposes a decision-tree approach based on clinical risk factors for abnormal outcomes in newborns with the neurological syndrome to assist in neonatal encephalopathy prognosis as a complementary tool to the acknowledged scoring systems. We retrospectively studied 188 newborns with associated encephalopathy and seizures in the perinatal period. Etiology and abnormal outcomes were assessed through correlations with the risk factors. We computed mean, median, odds ratios values for birth weight, gestational age, 1-min Apgar Score, 5-min Apgar score, seizures onset, and seizures duration monitoring, applying standard statistical methods first. Subsequently, CART (classification and regression trees) and cluster analysis were employed, further adjusting the medians. Out of 188 cases, 84 were associated to abnormal outcomes. The hierarchy on etiology frequencies was dominated by cerebrovascular impairments, metabolic anomalies, and infections. Both preterms and full-terms at risk were bundled in specific categories defined as high-risk 75–100%, intermediate risk 52.9%, and low risk 0–25% after CART algorithm implementation. Cluster analysis illustrated the median values, profiling at a glance the preterm model in high-risk groups and a full-term model in the inter-mediate-risk category. Our study illustrates that, in addition to standard statistics methodologies, decision-tree approaches could provide a first-step tool for the prognosis of the abnormal outcome in newborns with encephalopathy.

## 1. Introduction

Neonatal brain injury or neonatal encephalopathy (NE) is a significant morbidity and mortality factor in both preterm and full-term newborns. The incidence of NE is estimated in the range of 2.5 to 3.5 per 1000 live births, carrying an important burden for later neurological outcomes such as epilepsy, cerebral palsy, cognitive impairments and hydrocephaly [1]. Hypoxic-ischemic encephalopathy (HIE), cerebral hemorrhage (HC), infections (INF), metabolic abnormalities (METAB) are all severe etiologies in this complex clinical concept. At a molecular level, many pathways were incriminated in apoptosis of premyelinating oligodendrocytes or subplate neurons involved in perinatal brain development. Glutamate rising concentrations or free radical reactive species (both oxygen and hydrogen) in HIE, inflammatory cytokines such as TNF-α, IL-1b, IL-6, 12, 15, 18 from activated microglia and astrocytes and low pH in INF (including SARS-Cov2), free iron secondary to HC were extensively mentioned in both white and grey matter injuries. On a different scale, MRI images showed an association between white matter injuries and loss of the grey matter volume documented in both preterms and full-terms [2,3]. The most referred areas were in thalamus, basal ganglia, dentate cerebellar nuclei and hippocampus.

Many research reports associated specific MRI injury patterns with lower cut-off values for Apgar scores, birthweight and/or pH. More than two-thirds of the NE cases with brain injury occur near the birth time, and a prompt postnatal therapeutic intervention could alleviate the outcomes in newborns at risk [1,2,3,4,5,6]. Therefore, many scoring systems attempts based on a widely used model (the logistic regression) were proposed for different combinations of independent risk factors to assist in neonatal encephalopathy outcome prediction. Birthweight < 2800 grams, Gestational age < 37 weeks, Apgar scores < 7, ultrasound categories (different degrees from I to IV scale and different locations of hemorrhages, hydrocephaly, cerebral malformations) and MRI biomarkers (white and grey matter injuries), seizures onset < 24 h or >72 h, seizure duration values > 12 h, EEG pattern (moderate abnormal or severe abnormal) were the most frequent biomarkers analyzed in relation to abnormal outcomes [5,6,7,8,9,10]. Several studies associated specific MRI injury patterns with lower cut-off values for Apgar scores, Birthweight and/or pH, and lower or higher cut-off values for seizures onset. However, their distinctive significance levels as risk predictors depended on the studied population (full-terms or preterms) and the study designs. Consequently, different cut-off values were reported for the same biomarkers [7]. Moreover, the main outcomes in neonatal encephalopathy syndrome such as cognitive impairments, epilepsy, cerebral palsy, or hydrocephaly frequently overlap. Currently, the adverse outcomes for neonatal encephalopathy syndrome associating seizures are either studied distinctively for each etiology and each population (preterms or full-terms) or combined [5,6,7,8,9,10]. A recent meta-analysis performed by Glass et al., 2018 on neonates with brain injury both in preterm and full-term neonates associating seizures as comorbidities proposed a risk stratification strategy for both preterms and full-terms to forecast the outcomes based on the combination of the most frequently reported risk factors, to properly categorize the neonates at risk [10].

Consequently, our objective was to pursue a risk-stratification model by developing a decision-tree algorithm and subsequently a cluster analysis. The goal was to interlink the most accessible parameters in neonatology units worldwide, even in the low-income countries with poor resource settings. This algorithm would assist as a preliminary tool to forecast the outcomes in an integrated manner generating risk groups and their median profiles based on these risk factors. It could complement the acknowledged scoring systems based on logistic regression which typically employ the independent predictors. Considering the increasing reports of neonatal neurological impairments within the ongoing COVID-19 worldwide pandemic context such an approach would add importance in clinical practice [11,12]. Our model addressed both the preterm and term categories, irrespective of the etiology or the composite nature of the outcome (epilepsy, cerebral palsy, cognitive impairments, and hydrocephaly) as it was already suggested in the Glass’s meta-analysis. To date, the literature reporting other algorithmic tools than logistic regression is scarce and focused on specific etiology (HIE) and specific population (full-terms) [5]. Defining the risk groups in both neonatal categories (preterms and full-terms) would provide a fast preliminary evaluation by the health practitioners (pediatric neurologists or neonatologists) and the risk prognosis for the affected neonates. We considered all available predictors from the medical records examined. Etiology, gender, area (rural/urban), birth weight, gestational age, 1-minute Apgar score, 5-min Apgar score, ultrasound imaging markers, seizures onset, seizures duration monitoring and EEG patterns were studied as risk factors for an abnormal outcome in composite newborns populations (preterms and full-terms) with encephalopathy syndrome associating seizures as comorbidity to develop the model.

## 2. Materials and Methods

### 2.1. Patients and Methods

Our retrospective study included 188 newborns admitted between 1 January 1995 and 31 December 2005, to the Neonatal Compartment of Sibiu County Hospital and further evaluated in the Neurological Compartment of Pediatric Clinical Hospital from Sibiu, Romania. The data were examined according to the Declaration of Helsinki principles and the study was approved by the institutional ethics committee (ethical approval code 6475/02.11.2018). The analysis focused on both prematures and full-terms with associated neurological syndrome and seizures as comorbidities during the perinatal period. Medical data for each patient were reviewed regarding pregnancy, maternal pathology, gender, area (rural/urban), birth type, 1-min and 5-min Apgar scores, birth weight, gestational age, birth pathology, clinical exam logs and seizures’ treatment.

Other medical records, including laboratory results or imaging biomarkers (ultrasound or CT when available) were interpreted with respect to the etiology (HIE, HC, INF, METAB) and outcomes [7,8,9,10,13,14]. We categorized the available seizures recordings as subtle, clonic, tonic, myoclonic, generalized with or without EEG epochs confirmation [7,14,15]. Data regarding seizures onset, seizure duration monitoring, recurrences and electrical semiology were retrieved from medical charts. The records selection was performed by certified personnel. Recurrences mentioned in the records were considered in case of symptoms reappearance after 24 h of therapeutic control. We considered in our analysis the neurological sequels evaluated at the last follow-up during the first two years after birth [6]. Abnormal outcomes were assessed using standardized tests according to literature guidelines [6,8,16,17,18,19] (more details in Appendix A.1). The global outcome was defined as “abnormal”-1, if the patient had at least one of the following: epilepsy, cerebral palsy, one or more developmental domains delays and hydrocephaly and normal-0, otherwise.

### 2.2. Data Analysis

#### 2.2.1. Statistical Analysis

Numerical variables were assessed by calculating mean and median values, standard deviation, 95% confidence interval, minimum, maximum, interquartile range (IQR). Then, we applied the Student *t*-test on normally distributed data or the Mann–Whitney test on non-normally distributed data. For qualitative variables analysis, we estimated percentages. Fischer and chi-square (χ2)-tests were employed to study their association with outcomes (odds ratio-ORs, 95% confidence interval CI, *p*-value). Statistical significance level considered was 0.05. To our goal, we subsequently applied the decision-tree approach (CART) to forecast the abnormal outcome and cluster analysis to study the distribution of this outcome.

#### 2.2.2. Machine Learning Approach

Our analysis design was implemented in a pipelined fashion, first using descriptive statistics on the studied parameters, then CART and cluster analysis to extract and visualize specific risk groups.

Decision trees are a machine learning technique for classifying the data into categories, prospecting the hidden patterns within the data, as outlined in previous studies [20,21,22,23,24,25,26,27,28]. The resulting graph is a tree-like model of decisions considering the target variable, in our case the global outcome previously presented. It is generated backwards from the root at the top and branching until the split stops, interlinking all the predictors to forecast the global outcome. Branching is made on a condition (internal node) placed on the predictor variable to further split in the branches to reach a decision. The end of a branch is the “leaf”, the decision or the child node. The stopping criteria for growing the tree might be chosen as either longest path length from top root to a child node, or selecting a minimum number of training inputs for each child node.

We computed the CART models in pruning mode, considering different combinations of the risk factors previously studied using standard statistical methods. After the tree is grown to its full depth until stopping criteria are met, pruning trims the tree down (removing the nodes that provide less additional information) to the smallest subtree that has an acceptable risk value (more details are presented in Appendix A.3.1). CART pruned models perform cross-validation technique using cost-complexity approaches for trimming in order to minimize the average of the mean square prediction errors and to increase the stability of the model [21,24,25,27,28].

Only the models selecting predictors with the accuracy of more than 70% for abnormal outcomes forecast as a cut-off point of performance were kept for further analysis [5,20,21,22,23,24,25].

Subsequently, for the selected model, we explored in the form of prognostic rules for an abnormal outcome, the interdependencies between the risk factors selected by the algorithm. Then, we proceeded to refine our analysis by grouping the patients in low, intermediate or high-risk categories based on complications rates generated by CART decision tree [5].

Finally, cluster analysis was implemented at the end of the analysis pipeline as a profiling tool to highlight the significant differences of central tendencies values for the prognostic factors chain found in the low risk, intermediate and high-risk groups.

Cluster analysis is an unsupervised learning tool for clustering observations according to the similarity of their characteristics [21]. In our study, we used the Two-step Cluster Component algorithm, a combination of K-means cluster method and hierarchical cluster method, to generate, detect and select the groups based on both continuous and categorical data types (more details are presented in Appendix A.3.2).

To this goal, each case is added based on its similarity to existing nodes. Then, using the hierarchical cluster method, it clusters the sub-clusters from the previous step (leaf nodes of the CF tree). As a result, patients belonging to different clusters are different from each other, and patients belonging to the same cluster are similar, according to the grouping based on indicators scores. The number of clusters is selected in a two-stage approach cluster analysis based either on (AIC-Akaike Information Criterion or BIC-Schwarz’s Bayesian Criterion). This approach provides relevant differences when visualizing between and within the identified risk groups in CART output.

## 3. Results

For the studied period, in the Sibiu County, there were 48,377 deliveries (~4838/year) recorded in the neonatal departments (Sibiu, Mediaş and Cisnadie towns). During this period the medical records revealed 206 cases with neonatal seizures (121 full-terms and 85 preterms) of which 28 ended in death. A detailed presentation regarding pregnancy pathology, birth events and the treatment type for seizures is presented in Appendix A.2.

### 3.1. Etiology

From the total cohort of 206 cases, almost half of the remaining 188 cases, were associated with abnormal outcomes (Table 1). Cerebrovascular etiology, with hypoxic-ischemic encephalopathy, cerebral hemorrhage or both, was by far the most common cause of neurological sequels development. Furthermore, it should be emphasized that most of the infections ended in neurological sequels. The same pattern could be noticed for malformations and strokes, while in metabolic disorders, only a few cases had an abnormal outcome. Most of the cases often presented a combination of abnormal outcome types. Nevertheless, the distribution was dominated by the motor deficits followed by cognitive deficits, epilepsy, and hydrocephaly.

### 3.2. Demographics

In our retrospective cohort, the male gender cases were significantly higher (70.2% vs. 29.8%). Still, more than half (59.8%) did not show any complication. Conversely, in females, approximately the same proportion of patients (55.4%) had abnormal outcomes, with a 1.85 OR (*p* = 0.057) considering the males as reference. Urban, rural categories distribution (59% vs. 41.0%) showed a higher number of sequels in rural cases (49.4% with OR = 1.377, but not statistically significant *p* = 0.284).

#### 3.2.1. Gestational Age and Birthweight

Our data showed a prominent proportion of full terms (71.8%) versus preterms (28.2%), and a higher rate of complications was expected in prematures. In this category (<37 weeks), 58.5% of the cases presented sequelae. For comparison, in full-terms, an inverse percentage distribution of abnormal outcomes was recorded both in the 37–39 weeks category (34.9%) or >39 weeks (43.1%). Significantly lower gestational age (GA) and birth weight (BW) values were noticed in the abnormal outcome cases with 1.864 and 6.9 ORs, respectively (*p* = 0.089 and *p* = 0.000) taking GA > 39 weeks and BW > 2800 g as references. The computed means for GA (weeks) (38.29 ± 2.97-normal outcome vs. 36.27 ± 5.05-abnormal outcome, *p* = 0.005) and BW (grams) (3014.88 ± 636.92-normal outcome vs. 2668.63 ± 880.62-in abnormal outcome) further highlighted this observance (Table 2).

#### 3.2.2. Apgar Scores, Reanimation and Ultrasound Imaging

On the same note, the 1 min-APGAR score < 7 was more likely to increase the risk for an abnormal outcome (OR = 2.604, *p* = 0.005) however 5-min APGAR score < 7 showed only a tendency (*p* = 0.14) to a two-times increased risk (OR = 1.935). For both AS1 and AS5, the range between 8–10 were selected as a reference. This cut-off value (<7) was noticed in both 1-min Apgar Score (AS1) and 5-min Apgar Score (AS5) data and in the range of AS1 and AS5 means (Table 2). For the entire cohort, AS5 mean values were almost a unit higher than AS1 (*p* = 0.000). This difference was maintained in both normal and abnormal outcome cases (*p* = 0.000) (Table 2).

Reanimation procedures were documented in less than 20% of the study population (15.4%), of which 58.6% were sequelar. The rest of the patients (84.6%) had an inverted percentage proportion distribution of long-term clinical course (57.9%-normal outcome and 42.1%-adverse outcome). Then, 77.2% of the total cases had moderate (42.1%) and severe (35.1%) abnormal ultrasound findings. In both normal and moderate ultrasound anomalies categories, the normal (50%) and abnormal (50%) outcomes distribution was similar. In severe ultrasound anomalies (35.1% from the total cases) 77.5% cases had an abnormal outcome. Further analysis of categories (normal, moderate abnormal and severe abnormal—Appendix A.1) revealed 3.44 OR for an abnormal outcome only in cases with severe abnormal ultrasound changes compared to normal ultrasound cases considered as reference or to moderate anomalies ultrasound cases.

#### 3.2.3. Seizure Events

The most prominent seizure types associated with neurological complications were subtle seizures followed by clonic, tonic, myoclonic and generalized seizures. However, more than half of the cases presenting clonic, myoclonic and/or generalized seizure types had an abnormal outcome (Table 3).

Median values for seizures onset (ONS) were recorded after the first 24 h of neonatal life. Higher values were documented for the abnormal outcome instances, however not statistically significant. This was also the case for seizures duration monitoring (SDM) medians (Table 4). Again, a further analysis on the categorial approach drew attention to the tendency for the risk odds in adverse long-term outcomes evolution (58.3%) vs. normal outcomes evolution (41.7%) with 1.96 OR for ONS > 72 h (13.5% of the total cases) (*p* = 0.141). The 24–72 h interval (60.7% of the total cases) was taken as a reference. In both subcategories, ONS < 24 h (25.8% of the total cases) and ONS between 24–72 h, more than 55% cases (56.5% and 58.3% respectively) were distributed in the normal outcome group but not statistically significant (*p* = 0.834). Both SDM between 12–24 h and SDM > 24 h subcategories had an approximately 2 OR (2.058 and 1.871) for an abnormal outcome.

On the available EEG records, regardless of long-term clinical evolution, a case in two presented modified patterns. A thorough analysis of EEG traces correlated with long-term clinical course indicated that abnormal EEG epochs cases were 3.33 to 5 times more likely to develop an abnormal outcome depending on pattern severity (moderate or severe).

More than two-thirds of the patients had a medium form of encephalopathy (69.2% of the total cases) and the rest a severe form (30.8% of the total cases). It should be noted that from the medium form, over a half (59.5%) had a normal outcome while an inverted proportion was recorded in the severe form (57.1%) (*p* = 0.037, OR = 1.961).

### 3.3. CART and CLUSTER Analysis

**Figure 1:**
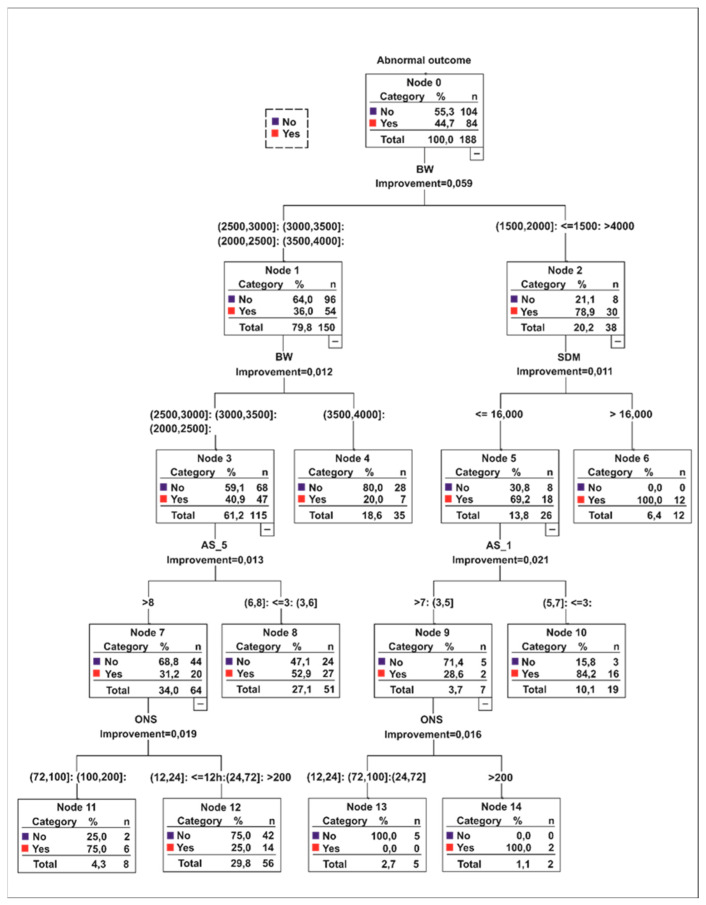
CART output illustrates a four-level decision tree (1–4). The cases partition is based on BW, AS1, AS5, ONS, SDM as prognostic factors related to abnormal outcomes (yes/no). The minimal change selection in impurity was 0.0001, whereas 5/3 were elected as minimal values for parent/child node. The hierarchy consisted of BW at level 1 and 2, SDM at level 2, AS1 and AS 5 at level 3, ONS at level 4.

The elected CART algorithm model classified the cases with an abnormal outcome using as risk factors five parameters of interest, namely BW, AS1 and AS5, ONS and SDM (Figure 1).

The overall accuracy for the CART analysis was 73.4% (75% for the abnormal outcome). We considered specific categories defined as high-risk 75–100% (nodes 6, 10, 11, 14), intermediate risk 52.9% (node 8) and low risk 0–25% (nodes 4, 12, 13).

In the high-risk category (38 cases), every node analysis on a case-by-case approach revealed interesting predictor combinations related to the etiology, in both term and preterm newborns. In term newborns for BW > 4000 g, there was an association of either an SDM > 16 h and a complex composite etiology (HIE + HC + INF, metabolic) or an SDM ≤ 16 h but with an AS1 < 3 (node 6, 10). In both full terms or late preterms, with BW ranging between 2000 g and 3500 g and AS5 > 8, the explanation for a potential abnormal outcome is etiology per se (HIE, HC, combined HIE + HC or AVC) with seizures as comorbidities with ONS > 72 h (node 11). In very or extremely preterms we found three instances: (1) BW ≤ 2000 g, SDM > 16 h and etiology dominated by HIE, HC, INF or a combination of them (node 6); (2) BW < 2000 g, SDM ≤ 16 h and AS1 5–7 (node 10); (3) BW < 1800 g, AS1 ≤ 5, GA ≤ 34 weeks, ONS > 200 h associated with the HIE + HC and INF etiology (node 14).

With regards to the intermediate-risk, 27 cases were classified accordingly. The BW ranged between 2000 and 3500 g and AS5 < 8 (node 8). Nonetheless, the algorithm could not specify any other clinical parameter to differentiate the cases.

**Figure 2:**
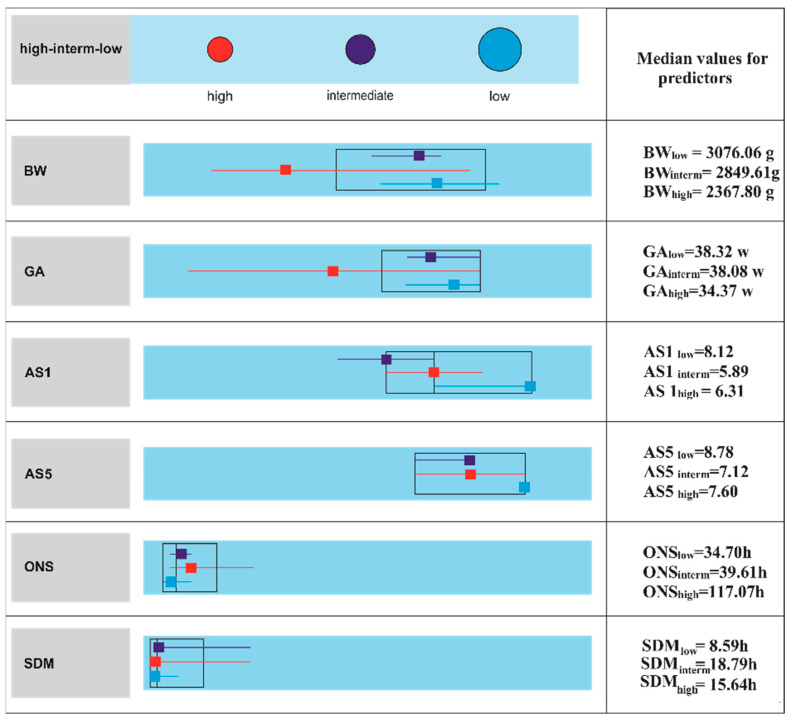
Cluster analysis for high-risk, intermediate and low-risk categories based on BW, GA, AS1, AS5, ONS, SDM. Median values for the predictors highlight the tendencies for each cluster. We recorded a fair silhouette measure of cohesion and separation for the three clusters (high, intermediate and low risk cases).

Further two-step cluster analysis on the nodes showed the median values for the predictor variables. An observation of great importance highlights the fact that in high-risk patients, the median values illustrated the tendencies towards a preterm newborn profile whereas in the intermediate-risk category towards the full-term. AS1 and AS5 presented slightly lower median values in full-term versus the preterm model for the risk groups. On the other hand, the SDM median in the high-risk group was almost twice as high as in the low-risk category but slightly lower than in the intermediate-risk group. Then, in the high-risk group, the median value for ONS was almost three times higher compared to median values in the low and intermediate-risk cases suggesting an important prognostic role for this parameter (Figure 2).

A synoptic insight regarding algorithms output brought forward a clustered clinical picture (Table 5). Both preterms and full-terms were distributed on risk categories based on etiology and specific predictors’ values. The smallest group of cases from the cohort was selected in the high-risk category considering the most incriminated etiologies (HIE, HC or their combination). The prominent distribution of preterms suffering from AVC and INF cases was also made in this class. Furthermore, the most composite outcomes from epileptic, cognitive delays, motor delays and hydrocephaly sequels were recorded in this risk group demonstrating the algorithms’ ability to link the predictors properly.

## 4. Discussion

The results confirmed several findings reported in other studies. Regardless of gestational age, the poor prognosis for neonatal encephalopathy came with etiology first [29]. Etiology pattern distribution was similar, although the percentages varied and were dominated by cerebrovascular etiology. Neonatal seizures comorbidities further increased the risk, especially in preterms and female gender. However, there were more male than female cases with neonatal seizures than in other reports [7,30,31,32,33,34]. Likewise, in agreement with the literature, our findings showed more numerous urban cases [35,36,37]. In reference to seizure semiology, we could not infer any conclusion for a specific seizure type role on the abnormal outcomes because many cases associated more than one seizure type. Consequently, as Pisani et al. suggested in their work, we did not input seizure semiology as a predictor in the CART algorithm [16]. Nevertheless, we noticed that in more than half of the cases with clonic, myoclonic and/or generalized seizure types, there was an abnormal outcome. Our results are in accordance with other authors’ findings regarding the most significant seizure types referred to as important risk factors, that could be associated with the abnormal outcome [8,16].

On the EEG background findings, our available records were similar to other reports regarding odds ratios and percentages for abnormal outcome in patients with moderately-severely abnormal patterns [6,7,8,16]. This was also the case for the cranial ultrasound. However, these variables showed prognostic sensitivity only within the univariate analysis. Even though the CART algorithm did link EEG background, cranial ultrasound with other predictors, we discarded these models because of the incomplete data and the consequent modest positive prediction performance [5,22,25].

In the outcome categories distribution, we found more numerous cases with motor and cognitive delays than epilepsy or hydrocephaly than in other reports. We believe the differences are based on study designs, cohorts and data consistencies as Lai et al. already suggested [8].

### 4.1. Preterms

Many research papers focused on age-specific neonatal populations to study etiology, risk factors and outcomes. In preterms, hemorrhage and infections were incriminated for most of the abnormal outcomes followed by HIE and stroke (focal ischemia) [38,39,40,41]. It is a known fact that neurological sequels can reach almost a double prevalence in preterm versus term neonates in primary HIE and HC [10,38,42,43,44]. Stroke in neonates is frequently associated with intrapartum complications, and HIE or INF per se are considered risk factors for stroke. As Al Yazidi et al. and Saliba et al. argued in their reports, this observation could render the outcomes in some of the cases from our cohort documented with a combination of etiologies (HIE and HC/HIE, HC and INF) [40,41]. The predictive model did classify them as high-risk patients. However, we noticed that not all preterms with serious etiology suffered an abnormal outcome which pleads for the importance of the independent predictors selected by the algorithm. The CART flowchart provided eloquent cut-off points for each independent variable at different levels revealing hidden patterns of association between them. BW had the highest importance to indicate preterms disabilities and should be linked to AS1, AS5, ONS and SDM values. Lower ranges of preterms BW (<2000 g) were linked with lower cut-off points for SDM (<16 h), AS1 (5–7 or <5) and with higher ONS (>200 h) or with higher SDM (>16 h). Preterms associating complex etiology and higher ONS (>72 h) were properly assigned in the high-risk group, even with higher ranges of BW (2000–2500 g) and AS5 (>8) suggesting the prediction importance of later seizures’ onset.

There are different opinions on low Apgar scores and higher ONS and SDM as sensitive biomarkers in preterm or late-preterm neonates along with lower ranges of BW. Low Apgar scores might be explained by physiological immaturity rather than newborn distress [45]. On the other hand, there were several reports pleading for Apgar scores as markers of severity correlating with MRI white matter injury. These findings were documented to be ten times more common in preterms than in term infants [39,46]. Regarding SDM predictor, some of the reports do not seem to correlate it with the outcome [7]. However, in preterm infants with cerebral hemorrhage or infections, longer SDM and later ONS were associated with a poorer outcome [10,44,47,48,49,50]. Our findings are in accordance with the latter studies. In a nutshell, for preterms classified in high-risk groups, lower BW, with later ONS and longer SDM might suggest serious outcomes. For this combination, we must draw attention to the median Apgar values (AS1-6.31, AS5-7.6) especially that they are higher than the medians in the intermediate-risk group (AS1-5.89, AS5-7.12). These values might provide preliminary cut-off points clinically relevant for practitioners, linked with longer ONSs and SDMs, and might be considered for further clinical validation to forecast the outcomes.

### 4.2. Full-Terms

Similarly, in full-term neonates, our findings are consistent with other research communications. Most of the authors mentioned HIE as a frequent cause of abnormal outcomes, especially HIE grades II, III [14,29,51]. Focal ischemia, cerebral malformations and metabolic disturbances follow in order. In HIE, white matter biomarkers suggesting injury on MRI diffusion images can occur up to several days after insult, pleading for perinatal brain injury rather than in utero acquired injury [9]. As in preterms reports, we noticed different opinions among the authors regarding the sensitivity of one predictor over another. Miller et al. advocated that both GA and Apgar Scores are good predictors [9]. Li et al. emphasized the importance of GA over BW, mentioning a strong correlation between GA and the severity of MRI biomarkers [52]. In their reports, Pisani et al. pleaded for BW as a more reliable measure than the anamnestic GA, which was also the case for our algorithm output [16]. Harteman et al. and Miller et al. found that lower values of AS5 were associated with MRI images with predominant watershed injury (8.5-Harteman and 5-Miller) and basal ganglia or thalami predominant injuries (7-Harteman and 4-Miller) [9,53]. It seemed that the extent of watershed anomalies is related to long-standing antenatal risk factors while the location of the injury in basal ganglia and thalamus with acute intrapartum risk factors. The outcome was prone to be influenced mainly by basal ganglia, and thalamic lesions and Apgar scores are currently considered sensitive predictors in this regard. Moreover, Garfinkle et al. indicated a greater than five odds ratios for an adverse outcome (*p* < 0.05) in the case of AS1 ≤ 3 and AS5 ≤ 5 [5]. Furthermore, in many papers, AS1 (<4) was independently associated with an adverse outcome [6,8,14,54]. To summarize, the lower AS1 and AS5 scores are, the higher risks for an adverse outcome [11,14,44,45,51]. Our findings agree with this conclusion. The median values for AS1 (5.89) and AS5 (7.12) profiled the full-term in the intermediate-risk group while the AS1 (<3) placed the cases in the high-risk group even for a BW > 4000 g. Analyzing other predictors importance, we noticed that patients with single etiology and ONS (>72 h) were placed in the high-risk groups even for BW ranging between 3000–3500 g and AS1 (>8). Different reports revealed earlier ONSs and longer SDMs correlating with a poorer prognosis on epilepsy and other neurological impairments [8,38,43,50]. In several HIE studies, the ONSs were mentioned within 24 h. Nevertheless, some of the authors documented time intervals varying from 8–36 h to several days [7,12,14,29,53]. In full-terms, we report median ONSs (>36 h) and median SDMs (>15 h) in both intermediate and high-risk groups. In conclusion, risk stratification strategy using our decision tree model, complementary to independently assessing each predictor importance through logistic regression models in different scoring systems, might offer some answers to the differences presented in the literature. Longer ONSs and SDMs could have an important prognostic value in both preterms and full-terms, however more studies on larger cohorts are needed to explore this finding.

### 4.3. Decision-Tree Approach

Many scoring systems based on regression models with different risk factors combinations have been proposed to assist in neonatal encephalopathy prognosis. In our study, in addition to standard statistical methodologies, we used a decision-tree approach which provides a graph easy to understand for the clinicians. We succeeded in developing a preliminary prediction model for neurological outcomes based on low, intermediate and high-risk categories working both on preterms and full-terms with encephalopathy. At a glance, on cluster chart, the medians of BW, GA, and particularly ONS and SDM set apart two tendencies: namely the preterms model within the high-risk populations and the full-terms model in the intermediate-risk segments with both preterms and full-terms distributed in all categories based on the predictors cut-off points. We highlight the importance of BW as the first predictor in the prognostic chain rule. Then, we suggest that lower values regarding AS1 and AS5 in intermediate and high- risk populations should be linked with BW profiles on the superior level and very importantly with higher ONS and SDM values further down on the decision tree path. Our risk stratification strategy using CART decision-tree is consistent with other authors recommendations despite the different study designs (e.g., only full-term neonates suffering from HIE) [5]. Moreover, in a recent review of different reports regarding the scoring systems outputs based on logistic regression, Glass and the coauthors [10] proposed a risk stratification approach on similar predictors combinations for both preterms and full-terms but with different ranges related to different study designs [55,56,57,58,59,60,61]. Nevertheless, we highlight that our patterns are congruent with the predictors’ behaviors in their metadata analysis for the high-risk groups, especially for BW and ONS. Hence, we emphasize the consistencies of our model behavior, its abilities to explore hidden patterns in the data and its future perspectives [10,55].

On the other hand, different results emerged in the descriptive analysis compared to decision-tree approach suggesting a more discriminative performance for the latter. CART included in the algorithm output also the parameters with the tendency of statistical significance on the standard statistical evaluation as previously mentioned in the literature [21]. Moreover, predictors’ median values were adjusted using decision tree methods and cluster analysis. To our knowledge, this approach has important elements of originality, especially related to the design and the goal. The literature is scarce in this respect. We found a few decision-trees reports to forecast preterm birth, neonatal jaundice, neonatal infections and a reference paper for predicting neurological outcomes in full-term neonates with encephalopathy using decision trees (CART) and logistic regression [5,62,63,64,65,66]. In the latter, though, the focus was only on a specific age group (term infants) and a specific etiology causing the encephalopathy (HIE).

There are, however, some limitations to our proposed model. Even though we present a single center experience, and our sample size and study design are comparable with other authors’ approaches [5,16], a larger sample size would strengthen the confidence and the generalizability for our results. Moreover, the model could be implemented as a preliminary tool to interlink the most accessible parameters (GA, BW, AS1, AS5, ONS, SDM) in neonatology units, especially in the low-income countries with poor resource settings, still, other variables might be further considered. We have excluded from the generated models those selecting also EEG and ultrasound patterns as independent predictors because of missing data in several subjects. Nevertheless, as Glass et al. 2018 suggested in their meta-analysis, outcomes forecasting using the proposed panels of risk factors continues to be challenging. Consequently, there is a need for further multicentric randomized controlled studies, with larger sample size considering also other biomarkers with complete datasets such as pH, EEG, ultrasound, CT, MRI imaging patterns, and treatment variables such as therapeutic neonatal hypothermia. This approach might lead to an increase in classification accuracy, a higher stability of the decision-tree algorithm and a more discriminative importance regarding the cut-off points values associated with different patterns of brain injury [10]. This way, the algorithm could be adapted to perform in more advanced intensive care neonatal units.

As a final point, our algorithm classification rate of 75% for abnormal outcome in pruning mode with its built-in cross-validation in cost-complexity trimming, was comparable to the reported results with similar sample size, complete datasets, using the standard CART approach and subsequent cross-validation technique [5,22,25,28].

## 5. Conclusions

We proposed a prognosis tool for validation by clinicians, linking the most common predictors available at the bedside to identify the newborns at risk. In our design, the most important feature was the distribution of both preterm and full-term cases in all of the risk groups (high, intermediate and low) clustered along median profiles as it was suggested in recent meta-analyses in the literature.

## Figures and Tables

**Table 1:** Cases distribution based on etiology and abnormal outcomes.

Categories	Total (N/P) ^b^	Outcome (N/P)		Abnormal Outcome Types ^a^ (N/P)			
		Normal	Abnormal	Epilepsy	Cognitive Delays	Motor Delays	HCEP
Patients	188	104 (55.3)	84 (44.7)	30 (16)	51 (27.1)	71 (37.8)	24 (12.8)
**Etiology**							
HIE	133 (70.74)	76 (57.14)	57 (42.86)	20 (15.04)	34 (25.56)	45 (33.83)	16 (12.03)
HC	11 (5.85)	4 (36.36)	7 (63.64)	3 (27.27)	7 (63.64)	7 (63.64)	1 (9.09)
INF	13 (6.91)	3 (23.08)	10 (76.92)	3 (23.08)	4 (30.77)	9 (69.23)	5 (38.46)
MALF	5 (2.66)	0 (0.00)	5 (100.00)	2 (40)	3 (60.00)	5 (100)	2 (40)
AVC ^c^	2 (1.06)	0 (0.00)	2 (100.00)	2 (100)	2 (100)	2 (100)	0 (0)
METAB	22 (11.70)	19 (86.36)	3 (13.64)	0 (0)	1 (4.55)	3 (13.64)	0 (0)
DRUG	2 (1.06)	2 (100.00)	0 (0.00)	0 (0)	0 (0)	0 (0)	0 (0)

Abbreviations—HIE, hypoxic ischemic encephalopathy; HC, cerebral hemorrhage; INF, infections; MALF, malformations; AVC, stroke; METAB, metabolic; HCEP, hydrocephaly; ^a^—most of the cases had a composite abnormal outcome; ^b^—N-total number/P-percentage; ^c^—focal ischemia.

**Table 2:** Descriptive statistics for GA, BW, AS1, AS5 variables in both normal and abnormal outcomes for the study group.

Variables (Number of Cases)	Total	Outcome		*p*-Value ^a^
		Normal	Abnormal	
	M ± SD (95%CI); MIN, MAX, IQR (MEDIAN)			
GA in weeks	37.39 ± 4.16 (36.79–37.99) 26–44 36–40 (38)	38.29 ± 2.97 (37.71–38.87) 27–44 37–40 (39)	36.27 ± 5.05 (35.17–37.37) 26–43 33–40 (38)	*p* = 0.005 ^b^
BW in grams	2860.17 ± 772.94 (2748.96–2971.37) 900–4450 2.350–3.490 (2997.5)	3014.88 ± 636.92 (2891.00–3138.74) 900–4225 2.615–3.530 (3085)	2668.63 ± 880.62 (2477.52–2859.73) 900–4450 1992–3260 (2810)	*p* = 0.003
AS1	7.16 ± 2.31	7.55 ± 2.18	6.66 ± 2.37	*p* = 0.005
	(6.81–7.51)	(7.11–7.99)	(6.11–7.21)	
	1–10	1–10	1–10	
	6–9 (7)	7–9 (8)	6–9 (7)	
AS5	7.99 ± 1.64	8.25 ± 1.40	7.68 ± 1.84	*p* = 0.082
	(7.67–8.30)	(7.88–8.62)	(7.15–8.20)	
	2–10	3–10	2–10	
	7–9 (8)	8–9 (9)	7–9 (8)	

Abbreviations: M, mean; SD, standard deviation; CI, confidence interval, MIN, minimum, MAX, maximum; IQR, interquartile range; ^a^—significance was assessed by Mann–Whitney test; ^b^—significance was assessed by Student *t*-test.

**Table 3:** Cases distribution based on seizure types in normal and abnormal outcomes.

Categories	Total (N/P) ^a^	Outcome (N/P)		Abnormal Outcome Types (N/P)			
		Normal	Abnormal	Epilepsy	Cognitive Delays	Motor Delays	HCEP
**Seizures types**							
Subtle	142 (75.53)	85 (59.86)	57 (40.14)	17 (11.97)	37 (26.06)	50 (35.21)	17 (11.97)
Clonic	87 (46.28)	42 (48.28)	45 (51.72)	19 (21.84)	30 (34.48)	35 (40.23)	12 (13.79)
Tonic	57 (30.32)	28 (49.12)	29 (50.88)	13 (22.81)	21 (36.84)	24 (42.11)	10 (17.54)
Myoclonic	41 (21.81)	21 (51.22)	20 (48.78)	11 (26.83)	13 (31.71)	18 (43.90)	10 (24.39)
Generalized	26 (13.83)	12 (46.15)	14 (53.85)	9 (34.62)	10 (38.46)	10 (38.49)	6 (23.08)

^a^-N-total number/P-percentage.

**Table 4:** Descriptive statistics for ONS and SDM variables in both normal and abnormal outcomes for the study group.

Variables (Number of Cases)	Total	Outcome		*p*-Value ^a^
		Normal	Abnormal	
	M ± SD (95%CI); MIN, MAX, IQR (MEDIAN)			
ONS in hours	55.95 ± 81.06	53.65 ± 83.01	58.84 ± 78.98	*p* = 0.467
	(43.95–67.94)	(37.08–70.20)	(41.14–76.52)	
	1–500	1–500	1–400	
	23–72 (29)	23–48 (28)	21–72 (32)	
SDM in hours	12.90 ± 19.98	11.38 ± 19.76	15.2 ± 20.28	*p* = 0.102
	(9.37–16.42)	(6.86–15.89)	(9.43–20.96)	
	1–96	1–96	1–82	
	2–12 (3)	2–10 (3)	2–24 (4)	

Abbreviations: M, mean; SD, standard deviation; CI, confidence interval; MIN, minimum; MAX, maximum; IQR, interquartile range; ^a^—significance was assessed by Mann–Whitney test.

**Table 5:** Distribution on etiology and outcomes based on risk group categories after CART and CLUSTER output analysis.

	High		Intermediate		Low	
	N (PT/FT)		N (PT/FT)		N (PT/FT)	
**Etiology**						
HIE	29 (13/16)	70.73%	35 (9/26)	68.63%	69 (18/51)	71.88%
HC	2 (2/0)	4.88%	5 (0/5)	9.80%	4 (0/4)	4.17%
INF	7 (7/0)	17.07%	2 (1/1)	3.92%	4 (1/3)	4.17%
MALF	0 (0/0)	0.00%	4 (0/4)	7.84%	1 (0/1)	1.04%
AVC	2 (0/2)	4.88%	0 (0/0)	0.00%	0 (0/0)	0.00%
METAB	1 (0/1)	2.44%	3 (0/3)	5.88%	18 (2/16)	18.75%
DRUGS	0 (0/0)	0.00%	2 (0/2)	3.92%	0 (0/0)	0.00%
**Outcome ^a^**						
Epilepsy	14 (5/9)	34.15%	11 (2/9)	21.57%	5 (1/4)	5.21%
Cognitive delays	23 (11/12)	56.10%	14 (2/12)	27.45%	14 (3/11)	14.58%
Motor delays	32 (19/13)	78.05%	23 (5/18)	45.10%	16 (4/12)	16.67%
HCEP	12 (8/4)	29.27%	9 (2/7)	17.65%	3 (1/2)	3.12%

Abbreviations: N, total number; PT, preterm; FT, full-term; ^a^—most of the cases had a composite abnormal outcome.

## Data Availability

Data available on request due to restrictions (privacy and ethical)

## Appendix A

### Appendix A.1. In Depth Analysis of the Subjects’ Records

We analyzed the neurological syndrome records referring to Sarnat classification criteria for moderate and severe encephalopathy (levels of consciousness-lethargic/stupor, spontaneous activity-decreased/no activity, primitive reflexes-weak, incomplete/absent, pupils-miosis/mydriasis, seizures presence/absence, heart rate-bradi/tachycardia, respiration pattern-periodic/apnea, muscle tone abnormalities-hypotonia/flaccid and posture-distal flexion/decerebrate) [5].

According to Lombroso and Holmes criteria, we interpreted EEG epochs from medical logs as normal, moderately abnormal with low voltage traces (10–50 µV), intermediate pattern, and delta-theta asymmetric waves and severely abnormal with inactive traces (<10 µV), spike-wave paroxistical bursts, suppression bursts [7]. EEG semiology was analyzed by a pediatric neurologist with expertise in neonatal EEG [6,67,68]. On ultrasound findings, we included in moderate anomalies category the small periventricular echo densities and intraventricular hemorrhages grades I and II. Severe anomalies were associated with thalamic, basal ganglia, white matter, cerebral cortex or intraventricular hemorrhages of III and IV grades, hydrocephaly, cerebral malformations [6,8,16].

The etiologies triggering neonatal encephalopathy were documented based on medical history, imaging diagnosis (ultrasound, CT or MRI when available), laboratory analysis, and placental pathology. We included both single or composite etiology cases considering the acknowledged causes to be involved in the pathophysiology of the neonatal neurological syndrome [6,7,8,16]:1. Hypoxic-ischemic encephalopathy with either antepartum and/or intrapartum risk factors for perinatal asphyxia in the case of fetal distress and a 5 min-Apgar score < 6) [8], 2. Cerebral hemorrhage and/or stroke (ultrasound, computed tomography-CT) [6,8,16], 3. Metabolic impairments (electrolytes deficits, hypoglycemia, inborn error of metabolism on laboratory analysis) [6,8], 4. Infections (laboratory analysis, positive cultures either on CSF or blood cultures) [6], 5. Cerebral malformations (ultrasound, CT or MRI when available, history of maternal exposure to infection, drugs use, toxins or trauma [8], 6. Drugs.

The neonates with a medical record of encephalopathy and seizures in the neonatal intensive care were further monitored as infants and toddlers by the pediatric neurologist based on the standardized institutional protocol. We considered in our analysis the neurological sequels evaluated at the last follow-up in the first two years after birth [6]. Abnormal outcomes were assessed at 1 month, 6 months, first year and second year of age using standardized tests based on the literature endorsement [6,8,17,18,19,69]:

(1) Epilepsy was categorized accordingly, based on the proposed guidelines of International League Against Epilepsy (ILAE). The infants presenting recurrent unprovoked seizures events were classified as epileptic.
(2) Cerebral palsy was clinically formulated as an early non-progressive motor deficit with objective changes in motor movements and posture on medical examination according to the guidelines provided by the Executive Committee for the Definition of Cerebral Palsy in 2005 [17].
(3) Hydrocephaly was defined based on working definition proposed by International Society for Hydrocephalus and Cerebrospinal Fluid Disorders and as an active distension of the ventricular system of the brain resulting from the inadequate passage of CSF from its point of production within the ventricles to its point of resorption into the systemic circulation [18,19].
(4) The developmental delay was defined based on the Practice Guidelines proposed by the American Academy of Neurology-Child Neurology Society Practice Guidelines. We looked for a significant delay, at least two standard deviations below the mean with standardized tests, affecting one or more developmental domains (global delay): Gross or fine motor, Speech/language, Cognition, Social/personal, Activities of daily living. We collected thru standardized questionnaires completed by the pediatric neurologist for each patient for the evaluations at 1 month, 6 months, first year and second year of life, based on Munich Functional Development Diagnostics standardized tests.

The global outcome was defined as “abnormal” if the patient had at least one of the following: epilepsy, cerebral palsy, one or more developmental domains delays and hydrocephaly.

### Appendix A.2. Pregnancy Pathologies and Seizures’ Management

Pregnancy pathologies associated with neonatal seizures were represented by imminent abortion/premature delivery (37 cases), respiratory infections (8 cases), urinary tract infection (9 cases), pregnancy induced hypertension (8 cases), twin pregnancy (4 cases), antepartum hemorrhages (2 cases), antepartum trauma (2 cases), hydramnios (1 case). Imminent abortion was more frequent in the preterms (25.9% cases) compared to full-terms (9.9% cases) (*p* = 0.004). Placental pathology has been documented in 4.35% of the cases. The most frequent category was placenta praevia (5 cases) followed by placental abruption (2 cases) and retroplacental hematoma (1 case). Two thirds of the cases were natural childbirths while the rest of them were cesarean. The most frequent pathology corelated with the birth event was nuchal cord (30 cases) followed by amniotic fluid aspiration (24 cases), forceps delivery (6 cases), umbilical cord prolapse (1 case), umbilical cord knots (1 case), mother hemorrhagic shock at delivery (1 case). Intrauterine growth restriction was noticed in 38 cases of full-terms neonates and in 10.6% of the preterms (*p* = 0.01).The hierarchy in descending order for the number of cases related to fetus status was the following: 1. acute fetal distress (100 cases), 2. chronic fetal distress with acute episodes (63 cases), 3. amniotic fluid aspiration (24 cases), 4. fetal chronic distress without acute episodes (17 cases), 5. hyaline membrane disease (22 cases), 9 macrosomes (9 cases).

The antiepileptic treatment administered in the acute phase consisted of phenobarbital (20 mg/kg iv loading dose and 3–4 mg/kg/day iv maintainance dose) as a first-line therapy and diazepam (0.1–0.3 mg/kg iv/ir up to 3 administrations/day) as a second-line. In the recurrences, phenobarbital treatment was maintained at 2–4 mg/kg/day. Out of 188 cases, 22 patients developed epilepsy requiring chronic antiepileptic treatment. For most of these cases (17) valproate monotherapy efficiently managed the condition. The rest of the cases in this subset required bitherapy or polytherapy (5) with valproate associated with one or more of the following drugs: phenobarbital, acetazolamide, lamotrigine, ACTH, topiramate, clonazepam.

### Appendix A.3. Mathematical Models for CART and CLUSTER Algorithms

#### Appendix A.3.1. CART Algorithm

CART is a binary splitting decision-tree using Gini index (Equation (A1)) and entropy rule for data partitioning based on predictor variables and nodes purity, from parent to child node. The best solution with the most significant increase in node purity is chosen from all the possible splitting ways. The process runs recursively until the stopping criteria are reached, or no reduction in node impurity is possible.

The purpose is to identify the best split point (cut-off value) for a predictor variable defined as the point that maximizes the splitting criteria based on Gini index (Equation (A1)), Twoing impurity measure in case of categorial variables or LSD (Least Squares Deviation) impurity measure in case of continuous variables. Then, the algorithm finds the best node split choosing the predictor that maximizes the splitting criterion. It produces the highest reduction in node impurity. Once the value of a variable is selected, the node is split in two, and the process is reiterated to each “child” node [21,22,23,24,25,70]

The process stops when no further gain can be made, or some pre-set stopping rules are met. The minimum change in improvement threshold is a user-specified level, usually set at 0.0001.

The Gini impurity measure at a node t is defined as:

(A1) $$i(t)=\sum_{k,l}P(k|t)P(l\;t)$$

where k,l (1, …, K)—index of the class, P(k|t)—probability of a case in class *k* given that it falls into node *t*. At node *t*, the best split s of the node *t* is chosen to maximize the splitting criterion.

CART decision tree has the capability to work with different data types and distributions, is robust to outliers, handling the missing values effectively through surrogate splits with its fully automated mechanism [21,22,23,24,25,70].

#### Appendix A.3.2. CLUSTER Algorithm

This approach identifies pre-clusters or groupings firstly, based on Euclidean (A2) or Log-likelihood distance (A3)–(A5) whether the variables are continuous or categorical, successively constructing a Cluster Features (CF) Tree [71,72,73].

(A2) $$d_{\mathrm{Euclidian}\;(B,C)}=\;\sqrt{{\left(X_{B}-X_{C}\right)}^{2}+{\left(Y_{B}-Y_{C}\right)}^{2}}$$

(A3) $$d_{\left(m,n\right)}=\xi_{m}+\xi_{n}-{\;\xi }_{\langle m,\;n\rangle }$$

(A4) $$\xi_{s}=-N_{s}(\sum_{p=1}^{K^{A}}\frac{1}{2}\log({\hat{\sigma }}_{p}^{2}+{\hat{\sigma }}_{\mathrm{rp}}^{2})+\sum_{p=1}^{K^{B}}{\hat{E}}_{\mathrm{rp}})$$

(A5) $${\hat{E}}_{\mathrm{rp}}=-\sum_{l=1}^{L_{p}}\frac{N_{\mathrm{rpl}}}{N_{r}}\log(\frac{N_{\mathrm{rpl}}}{N_{r}})$$

where B, C are 2 elements from the data input in the Equation (A2), d (m, n) represents the distance between clusters m and n in Equation (A3); <m, n> are the indexes indicating the cluster generated from combining clusters m and n combination; KA-stands for continuous variables total number, while K B -for categorical variables total number; Lp-defines the categories’ number for the p-th categorical variable (A4); Nrpl represents the records number in cluster r whose categorical variable p takes l category; Npl represents the records number in categorical variable p that take the l category; ${\hat{\sigma }}_{p}^{2}$—represents the estimated variance (dispersion) of the continuous variable p, for the entire dataset; ${\hat{\sigma }}_{\mathrm{rp}}^{2}$—defines the estimated variance of the continuous variable p, in cluster n.
